# A semantic proteomics dashboard (*SemPoD*) for data management in translational research

**DOI:** 10.1186/1752-0509-6-S3-S20

**Published:** 2012-12-17

**Authors:** Catherine P Jayapandian, Meng Zhao, Rob M Ewing, Guo-Qiang Zhang, Satya S Sahoo

**Affiliations:** 1 Division of Medical Informatics, School of Medicine, Case Western Reserve University, Cleveland, OH 44106, USA; 2 Center for Proteomics and Bioinformatics, School of Medicine, Case Western Reserve University, Cleveland, OH 44106, USA

## Abstract

**Background:**

One of the primary challenges in translational research data management is breaking down the barriers between the multiple data silos and the integration of 'omics data with clinical information to complete the cycle from the bench to the bedside. The role of contextual metadata, also called provenance information, is a key factor ineffective data integration, reproducibility of results, correct attribution of original source, and answering research queries involving "**W**hat", "**W**here", "**W**hen", "**W**hich", "**W**ho", "Ho**w**", and "**W**hy" (also known as the **W7 **model). But, at present there is limited or no effective approach to managing and leveraging provenance information for integrating data across studies or projects. Hence, there is an urgent need for a paradigm shift in creating a "provenance-aware" informatics platform to address this challenge. We introduce an ontology-driven, intuitive Semantic Proteomics Dashboard (**SemPoD**) that uses provenance together with domain information (semantic provenance) to enable researchers to query, compare, and correlate different types of data across multiple projects, and allow integration with legacy data to support their ongoing research.

**Results:**

The SemPoD platform, currently in use at the Case Center for Proteomics and Bioinformatics (CPB), consists of three components: (a) Ontology-driven Visual Query Composer, (b) Result Explorer, and (c) Query Manager. Currently, SemPoD allows provenance-aware querying of 1153 mass-spectrometry experiments from 20 different projects. SemPod uses the systems molecular biology provenance ontology (SysPro) to support a dynamic query composition interface, which automatically updates the components of the query interface based on previous user selections and efficientlyprunes the result set usinga "smart filtering" approach. The SysPro ontology re-uses terms from the PROV-ontology (PROV-O) being developed by the World Wide Web Consortium (W3C) provenance working group, the minimum information required for reporting a molecular interaction experiment (MIMIx), and the minimum information about a proteomics experiment (MIAPE) guidelines. The SemPoD was evaluated both in terms of user feedback and as scalability of the system.

**Conclusions:**

SemPoD is an intuitive and powerful provenance ontology-driven data access and query platform that uses the MIAPE and MIMIx metadata guideline to create an integrated view over large-scale systems molecular biology datasets. SemPoD leverages the SysPro ontology to create an intuitive dashboard for biologists to compose queries, explore the results, and use a query manager for storing queries for later use. SemPoD can be deployed over many existing database applications storing 'omics data, including, as illustrated here, the LabKey data-management system. The initial user feedback evaluating the usability and functionality of SemPoD has been very positive and it is being considered for wider deployment beyond the proteomics domain, and in other 'omics' centers.

## Background

Though many molecular system biology research centers now have significant infrastructure in terms of instrumentation to acquire 'omics datasets, most of these datasets end up in study-specific data silos. Specifically, more than 50% of data being generated in laboratories are stored in local lab servers [1], which not only reduces data utilization and re-use, but also is a significant waste of funding resources [2]. In addition, the size of experiment datasets continues to grow; more than 48% of respondents to a recent *Science *journal survey regularly generate 1 GB (gigabyte) or larger dataset [1]. Therefore, there is an urgent need to effectively organize the data, cross-link the datasets across 'omics and clinical studies as part of the translational research roadmap, facilitate integration with legacy data, and allow seamless query across different types of data to gain research insight and accelerate research [2]. Proteomic studies typically make use of multiple different work-flows that provide information at different scales. For example, protein profiling allows for large-scale analysis of protein expression whereas interaction proteomics focuses on specific protein complexes or networks. The objective of this work is to provide a means of integrating data across proteomics studies and workflows to provide a more global view of the biological problem being studied. In addition, the primary proteomics data should be integrated with resources that provide annotation information such as protein function and pathways. For example, a researcher might acquire large-scale proteomics data from tumors of 30 patients corresponding to several different clinical stages of colorectal cancer and would like to answer questions such as:

### In which subset of patients and/or clinical stage is signaling pathway X most activated?

Extending the above scenario, the researcher may consider that although activation of pathway X is altered in a mouse model of disease Y, it is not clear that this is also the case in humans. Thus, if the researcher acquires datasets from several different cohorts of patients with disease Y, she might ask:

### Is the cognate pathway X also important in human?

At present, there is no informatics infrastructure in the CPB that is capable of supporting these categories of queries. In addition, the lack of an effective query platform is also a key reason that once the 'omics data has been acquired, analyzed and interpreted, the data is typically archived and serves no further process. This is important issue both in terms of maximizing the return on research funding and also ensuring that the value of 'omics data can be significantly increased if that data is carefully integrated into a growing corpus of data that can then be re-used in different contexts. For example, a researcher with a long-standing interest in disease X has acquired multiple large-scale proteomics and transcriptomics datasets over several years. In response to a newly published finding that Single-Nucleotide Polymorphism (SNP) in gene Y are associated with disease X, the researcher now wants to query all of her legacy data and ask

### Are there any new associations between gene Y and disease X?

In general, these types of queries are difficult to perform because they integrate several types of information, including biological annotations from outside sources.

## Provenance-aware integrated query environment

The role of contextual metadata describing the experimental conditions, for example sample type, instrumentation, sample preparation, and statistical measures, is being increasingly noted as a key factor in managing translational research data [2]. Contextual metadata is also called provenance information, derived from the Latin word *provenire *meaning the origin or history of data. Provenance metadata supports integration of comparable datasets, facilitates correlation of data across projects, and also supports analyses of data by answering "**W**hat", "**W**here", "**W**hen", "**W**hich", "**W**ho", "Ho**w**", and "**W**hy" queries (also known as the **W7 **model) [3,4]. Provenance has long been used in many domains to track the ownership of cultural artifacts and also in scientific research [5-7]. Traditional translational informatics tools have either ignored the role of provenance to the detriment of data quality or used it for basic operations (e.g. file versioning).

In addition to incorporating provenance metadata in medical informatics platforms, there is a well-recognized need for an intuitive and powerful query interface that can be directly used by researchers. Frequently, analysis and querying of 'omics datasets requires expertise that may not be available to many translational laboratories. For example, in a recent survey in the Science journal about 57% of researchers have either no support for data analysis or are dependent on others for managing experiment data [1]. Hence, there is a clear need to combine the query environment with the provenance-aware data integration platform to enable researchers to use contextual information to query and compare datasets using explicitly defined experiment conditions. In addition, the query environment should demonstrably reduce the technical complexity for query composition through use of visual interactive interfaces that transparently query distributed data, allow users to store query results for future reference, and show results in an intuitive manner [8].

The SemPoD platform is designed to address these challenges through use of provenance informationintegrated with a visual, ontology-driven, integrated query environment.

## Methods

We use two principal proteomics workflows used in the CPB as exemplars to describe the design and implementation of SemPoD, namely:

1. The first workflow is affinity-purification mass-spectrometry (AP-MS) workflow that enables the identification of specific protein complexes, thus identifying proteins that are associated with one another.

2. The second workflow is the shotgun expression proteomics that identifies and quantifies proteins in an unbiased manner from cells or tissues of interest.

Together, these two workflows account for approximately *50% of all experiments performed in the CPB *and have been used in approximately 20 separate projects, *generating over 3 Terabytes (TB) of data*.

**Figure 1:**
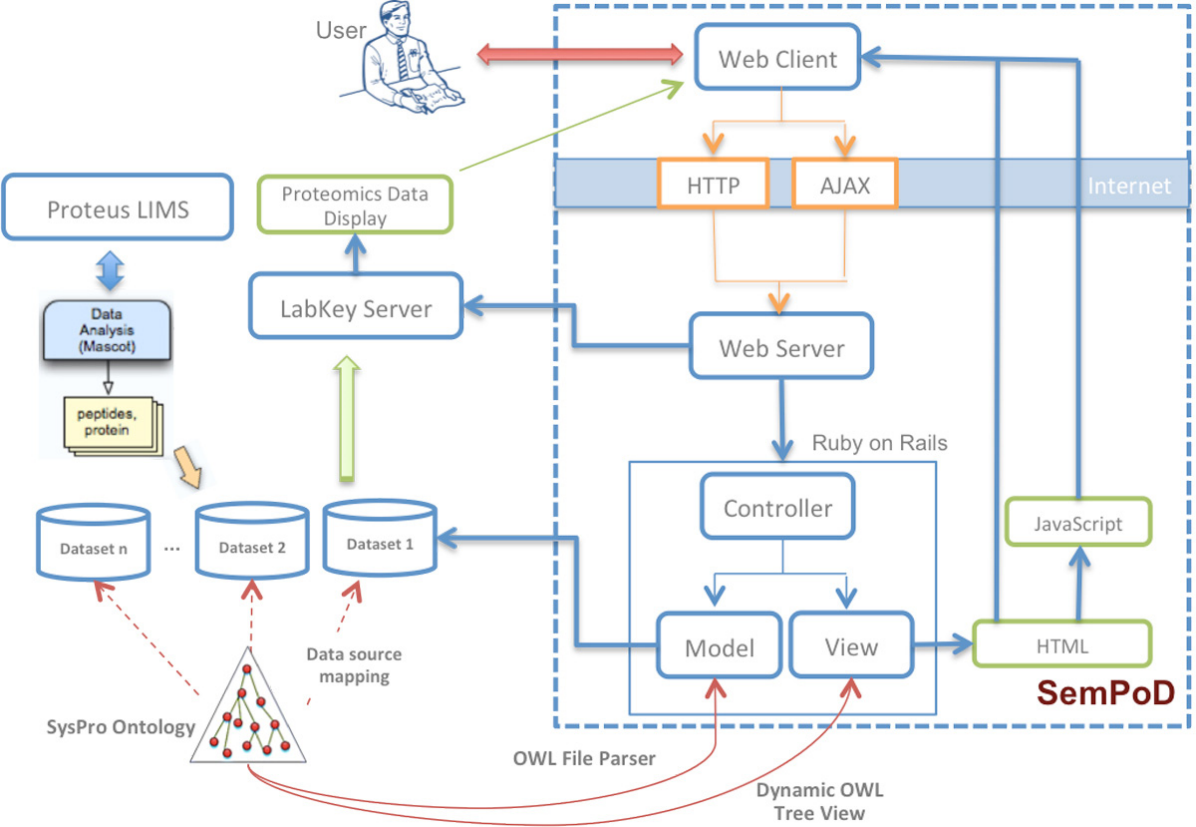
**The SemPoD Architecture with the SysPro ontology**. Figure shows the high-level architecture of SemPoD web-based dynamic query interface that uses the Model-View-Controller (MVC) architecture design pattern. The figure shows how SemPoD integrates 'omics datasets from heterogenous data sources like Proteus LIMS using a ontology-driven, provenance based approach. The figure also shows how SemPoD interfaces with data analysis and viewing applications like Labkey.

SemPoD was developed using agile software engineering methodology for rapid and iterative development in close consultation with the users. The agile engineering approach was combined with the Ruby-on-Rails web development framework that uses a Model-View-Controller (MVC) architecture pattern. The MVC pattern involves a strict separation of the application logic from the user interface, which allows SemPoD to seamlessly adapt to changing requirements of translational research studies, with a consistent query environment (Figure 1 illustrates the SemPoD architecture).

SemPoD leverages the SysPro ontology as the core resource to support various query functionalities, including "smart filtering" for reducing user effort in composing complex query patterns.

### The systems biology provenance (SysPro) ontology

At present, the provenance metadata associated with the different stages of the proteomics workflow at CPB is not collected in a systematic manner. Often, the provenance metadata is stored as hand-written notes in a lab book and is not immediately available for query and analysis of the proteomics dataset. Further, any modification in the experiment protocols or related experiment metadata information makes it difficult to correlate or integrate data from previous runs with new datasets. The use of a variety of terms to describe provenance increases terminological heterogeneity across different projects and makes it difficult to effectively integrate datasets.

Hence, the SysPro ontology was developed to model experiment metadata by re-using and extending existing minimum information reporting guidelines defined by the 'omics community. Several "minimum information" reporting frameworks have been developed and are now part of the minimum reporting guidelines for biological and biomedical investigations (MIBBI) project [9], which facilitates collection and representation of experiment metadata in a variety of scientific domains. The minimum information required for reporting a molecular interaction experiment (MIMIx) framework [10] is part of the MIBBI project and extends the minimum information about a proteomics experiment (MIAPE) [11] framework with additional metadata terms describing interaction information that are used in the experiment workflows at the CPB. Concepts and terms already described in MIMix, for example "interaction detection method", "co-immunoprecipitation" were used as initial concepts in the construction of the SysPro ontology. Further, additional proteomics workflow specific terms were added to SysPro to reflect the specific requirement of provenance modeling in CPB by extending the World Wide Web Consortium (W3C) PROV ontology (PROV-O) [12].

**Figure 2:**
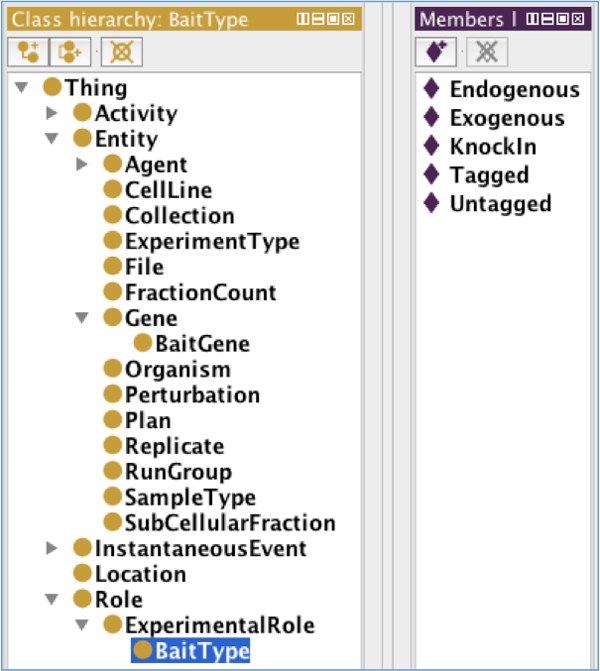
**The SysPro class hierarchy and instances for class 'Cell Line'**. This figure shows the hierarchy of classes in the SysPro ontology. This figure is a screenshot taken from Protege tool that was used to create the SysPro ontology. On selecting a class, for example 'Bait Type', the instances of this class is shown on the right pane, namely 'Endogenous', 'Exogenous', 'KnockIn', 'Tagged' and 'Untagged'.

The PROV-O is a reference ontology being created by the W3C provenance working group to facilitate provenance interoperability with a set of common provenance-specific classes and relationships. The PROV-O terms can be extended by various domain-specific applications, such as SemPoD [12]. The PROV-O consists of three primary classes namely, (1) prov http://www.w3.org/ns/prov . Activity that models processes occuring over a period of time, (2) prov:Entity that models resources that are described in provenance assertions, and (3) prov:Agent that represents specific type of prov:Entity or prov:Activity that are responsible for actions associated with prov:Activity. The PROV-O ontology classes are linked together with named relationships, such as prov:used, prov:wasAttributed, which allows effective modeling of provenance assertions, for example cell culture used an "endogeneous" bait type. The SysPro ontology extends the PROV-O classes and relationships to model provenance metadata associated with the AP-MS and shotgun expression proteomics workflows. Figure 2 illustrates the class hierarchy and "instance" values of the class "BaitType" in the SysPro ontology.

The SysPro ontology also facilitates cross-linking of 'omics data with a variety of related genomics and clinical datasets, which are annotated with domain ontologies [13]. A rapidly increasing number of biomedical domains, such as genetics, infectious diseases, and cancer, have created ontologies to model their domain information. These domain ontologies have significantly enhanced the use of standardized terminology across these communities. The most notable example is the case of Gene Ontology (GO) that is widely used to consistently annotate gene related information across a variety of applications [14].

**Figure 3:**
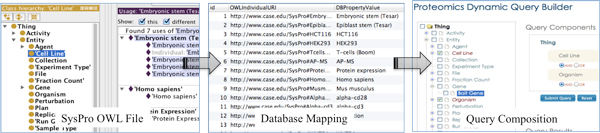
**The mapping process of SysPro ontology terms in the LabKey**. This figure shows the steps for configuring a data source for querying the underlying 'omics data. A data source can be dynamically configured by mapping the SysPro ontology classes to the underlying database. After configuration, a query can be composed using the query builder and submitted on this data source.

To allow experiment data generated in CPB to be linked to external datasets at UniProt (for protein data) and GeneDB, inter-ontology mappings between SysPro, GO, and the Protein Ontology (PRO) [15] can be semi-automatically created enabling SemPoD to support queries across both internal and external datasets. Currently, SemPoD uses mappings between the SysPro ontology and the underlying proteomics databases for query translation and execution. Figure 3 illustrates the mapping process from the CPB protomics database and SysPro ontology. The SysPro ontology allows SemPoD to not only adapt the functionality of the query environment according to user input, but also improve the performance of SemPoD query modules.

### The SemPoD query environment

**Figure 4:**
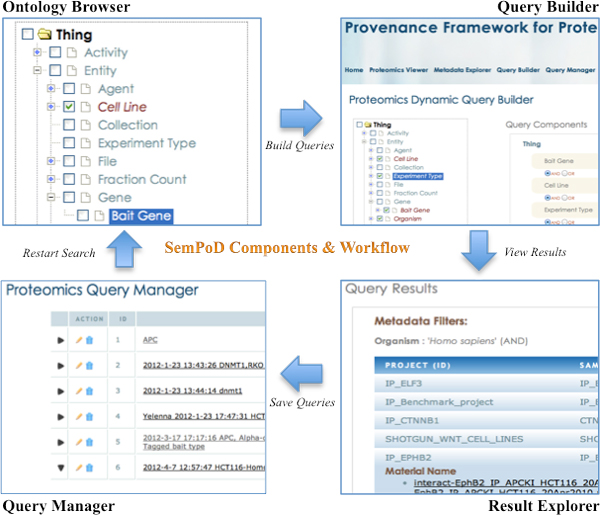
**The four constituent modules of SemPoD**. This figure shows the four main components of SemPoD namely the hierarchical ***ontology browser ***with checkboxes for selection of multiple concepts that will used as parameters in query composition, ***query builder***, ***results viewer ***that interfaces with third-party data analysis applications like Labkey and query manager that shows the list of saved queries that act as templates for future querying.

SemPoD consists of four main components, namely (1) the SysPro ontology browser, (2) the integrated query builder, (3) the result explorer, and (4) the query manager (Figure 4).

### SemPoD ontology browser and query builder

**Figure 5:**
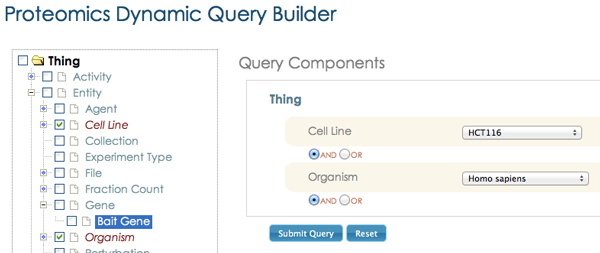
**SemPoD Query Builder**. Query Builder is an intuitive interface that allows selection of query conditions from the SysPro ontology browser and create dynamic queries by selecting different logical connectives and parameter instances.

**Figure 6:**
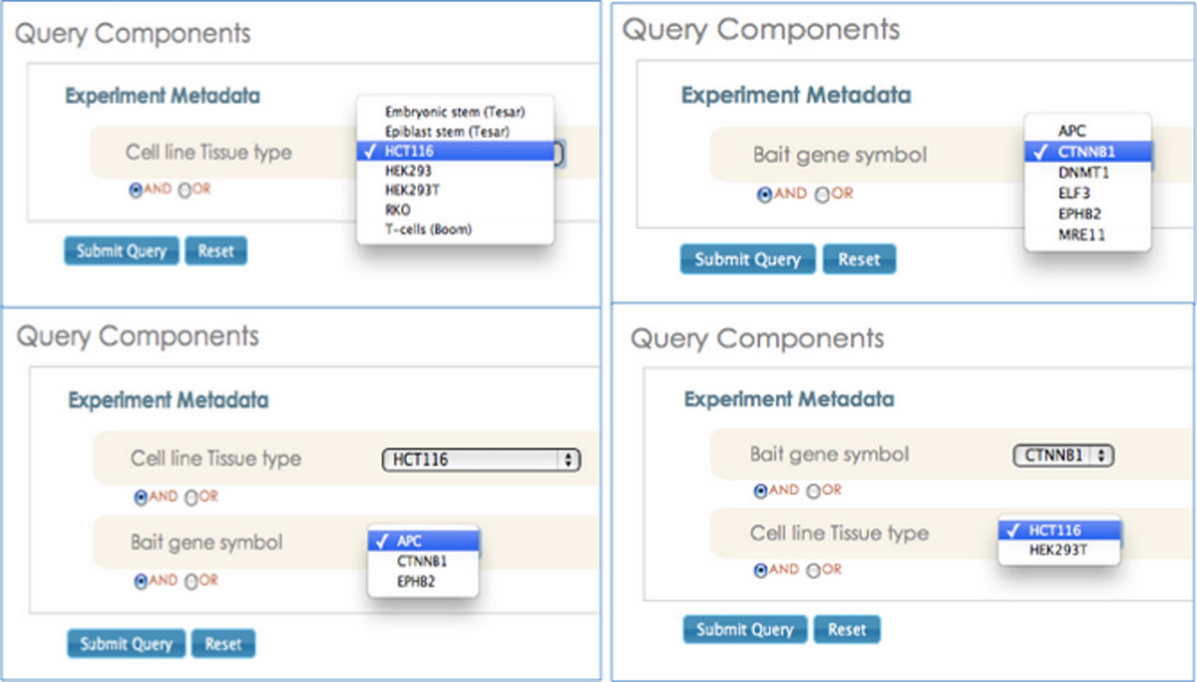
**Screenshot illustrating the "smart filtering" feature implemented in the query builder**. Smart filtering is an feature that enables effective selection of query parameter and their instances during query composition. Smart filtering updates the drop-down list for a selected query condition based on all the previously selected query parameters. The advantage of this approach is to eliminate selection of query parameters that will not bring any valid results.

The SemPoD query builder component (Figure 5) is an intuitive and flexible interface that allows researchers to directly browse the SysPro ontology class hierarchy and select appropriate terms to interactively compose expressive queries. Once a SysPro ontology class is selected by the user, the query composer automatically populates the the "drop-down" menu corresponding to the class, which allows the user to easily select specific value. For example, if an user selected the class "Cell line", the coressponding drop-down menu is populated with its "instance" values (Embryonic stem, Epilast stem cell or HCT116) as illustrated in Figure 6. Further, the users can compose complex query patterns by linking query terms with binary logical connectives("and", "or").

The SemPoD query builder uses the SysPro ontology to support an advanced feature called "smart filtering" that dynamically updates the query interface in response to previous user selections. Figure 6 illustrates this feature, with selection of two classesnamely, "Cell line" and "Bait gene" and the corresponding drop down menus that are automatically populated with instance values of the classes defined in the SysPro ontology. The "smart filtering" approach allows the users to quickly compose large query patterns by significantly reducing the time needed to search and locate appropriate values in the query builder interface.

**Figure 7:**
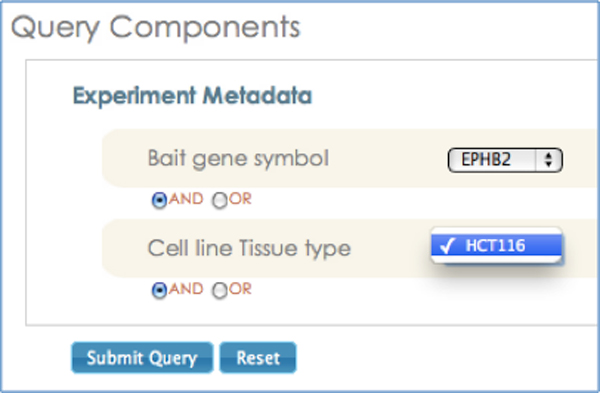
**Screenshot illustrating the use of property linking two instances for populating drop-down menus**. Smart Filtering feature leverages instance-level relationships defined in SysPro ontology, which links only specific instance values with each other.

**Figure 8:**
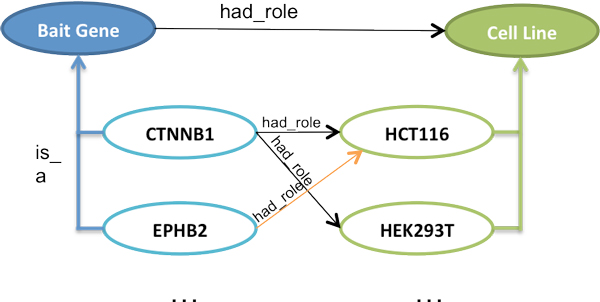
**Use of property hadRole in the SysPro ontology linking classes Cell Line and Bait Gene**. This figure shows an example of instance-level relationships between the Bait Gene and Cell Line classes defined in SysPro ontology.

Further, the "smart filtering" feature leverages instance-level relationships defined in the SysPro ontology, which links only specific instance values with each other. For example, the "EPHB2" instance of class "Bait gene is associated with only "HCT116", which is an instance of class "Cell line". Hence, when the user updates her selection of "bait gene symbol" from "CTNNB1" to "EPHB2", the corresponding instance value for the "Cell line" is automatically updated to "HCT116" (Figure 7). As discussed in the previous section, the SysPro ontology re-uses the PROV-O relationships to link both classes and instances reflecting domain-specific information in systems molecular biology. Figure 8 illustrates the use of "prov:hadRole" to link the "Bait gene" and the "Cell line" classes and their instances.

### SemPoD result explorer

**Figure 9:**
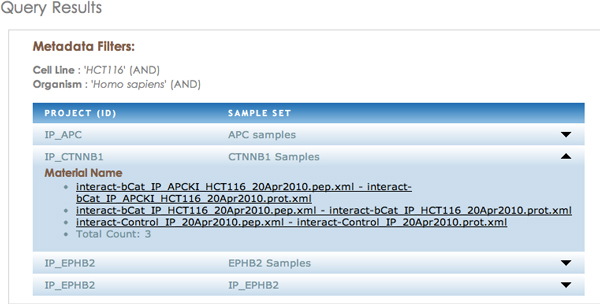
**The result explorer allows users to link out to the underlying Labkey database**. Query results are shown in separate tabs, one for each project. The experiment files are listed which then interface with underlying Labkey proteomics data.

The user can explore the results of their queries in the SemPoD result explorer (Figure 9), which lists the projects datasets that correspond to the experiment metadata criteria used in the query pattern. In addition, the result explorer links directly to the underlying LabKey proteomics data browser [16], which is used in CPB to store the results (after login credential have been initially verified). The seamless interface with the LabKey allows SemPoD to build on existing data management platforms that are already in use by many 'omics' centers without having to re-implement many features that already present.

### SemPoD query manager

**Figure 10:**
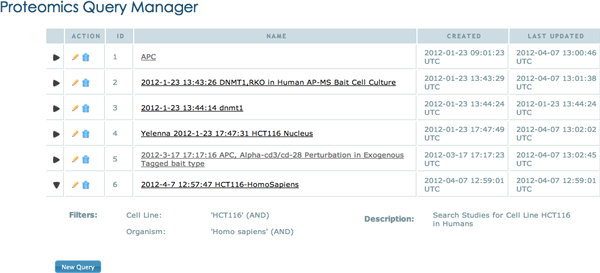
**The query manager showing a list of queries with details describing date of creation and update**. Query Manager shows the list of saved queries by differnet users. On expanding the row, the details of the query are shown.

The user can also save their queries using the 'Save Query' button in the query builder interface(Figure 6). A query name and description can be given to identify the query for later use. Figure 10 showsa screenshot of the query manager with a list of all saved queries. An user can select a specific query from the query list, view the query pattern, and re-execute the query if needed. The ability to store commonly used query patterns that can be retrieved later and also shared with other researchers is an important feature of SemPoD and has received positive feedback from users at CPB.

## Results

SemPoD has been deployed at the CPB and has been in use for over 2 months. SemPoD was evauluated both in terms of systematic user survey and scalability for queries with different levels of complexity over increasing size of data.

### User evaluation

**Figure 11:**
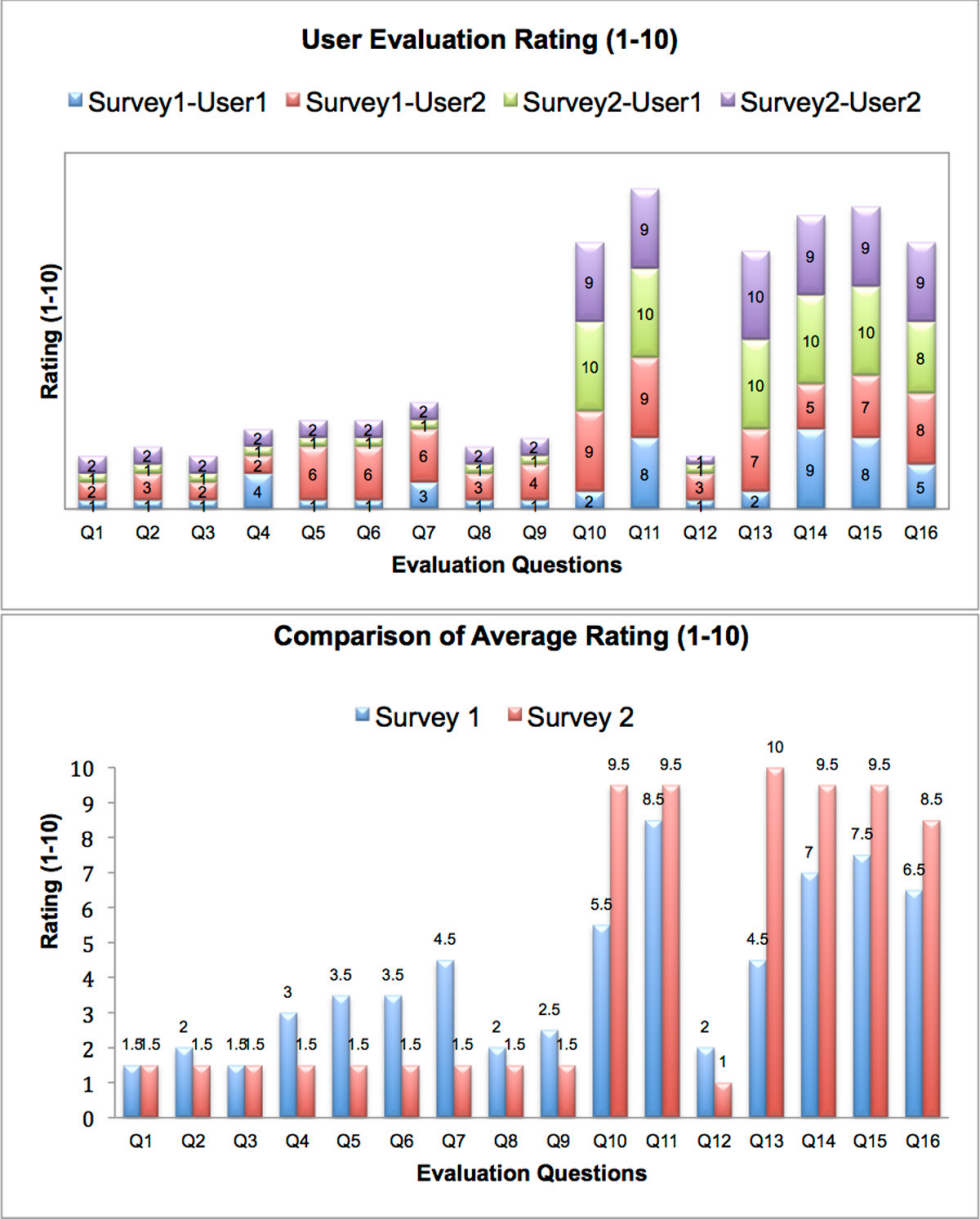
**Results of user feedback after 2 months and 4 months of SemPoD deployment**. User ratings for 2 surveys are shown for questions 1-16. Survey 1 was done after 2 months of deployment and survey 2 was done after 4 months of deployment.

The user evaluation was done in two-phases over a 4-month period, the first user feedback was collected after 2 months of deployment, and a second survey was conducted 4 months after initial deployment. The user feedback from each phase was used to update SemPoD and modified features were also evaluated in the subsequent user survey. The user survey consisted of 16 queries evaluating different aspects of SemPoD, including ease of query composition, navigability of the SysPro ontology terms, accuracy of result datasets, and presentation of data in the result explorer. The survey used a scale of 1-10 to measure response. For questions Q1-9 and Q12, the user response was recorded as a measure of "the difficulty level of query composition" with 1 representing "not difficult at all" and 10 representing "very difficult". For questions Q10, Q11 and Q13-16, the user response was recorded as a measure of "how informative, consistent, easy-of-use" with 1 represented "not useful at all" and 10 representing "very useful". Figure 11 illustrates the user feedback for the 16 survey queries, where "survey 1" refers to the first set of user feedback at end of 2 months and "survey 2" represents user response after 4 months. There is a significant increase in the positive response from survey 1 to survey 2, indicating an overall positive feedback and increased use of the tool for their research purposes.

### SemPoD scalability evaluation

In addition to user evaluation, the scalability of SemPoD was also evaluated with respect to increasing complexity of queries, in terms of logical connectives used to compose the query, and sizes of data. Table 1 lists the sets of queries used for the scalability evaluation over two datasets of size 20 GB and 50 GB respectively.

**Table 1:** Details of queries used to evaluate the scalability of SemPoD

QUERY DESCRIPTION	METADATA TERMS IN QUERY PATTERN
Q1. Search proteomics experiments in any human sample	Organism = 'Homo Sapiens'

Q2. Search proteomics experiments for 'Embryonic stem' cell line in any human sample	Organism = 'Homo Sapiens' (OR) Cell Line = 'Embryonic stem'

Q3. Search proteomics experiments for Human samples with Cell Line 'Embryonic stem' or Pertubated with 0 Dosage in Cytosol Subcellular Fraction	Organism: 'Homo sapiens' (AND) Cell Line: 'Embryonic stem' (OR) Perturbation: 'Dose = 0' (OR) Subcellular Fraction: 'Cytosol'

Q4. Search Experiments for Bait Gene 'DNMT1' in AP-MS experiments or WNT3A perturbations in Bait Run Group for Cell Line RKO	Bait Gene Symbol = "DNMT1" (AND) Experiment Type = "AP-MS" (OR) Perturbation = "WNT3A" (AND) Run Group = "Bait" (OR) Cell Line = "RKO"

Q5. Search Protein Expression Experiments for T-cells Cell Lines for Drosophila melanogaster organism perturbed with 10 ng in treated cell cultures	Experiment Type: 'Protein Expression' (OR) Cell Line: 'T-cells (Boom)' (AND) Organism: 'Drosophila melanogaster' (AND) Perturbation: '10 ng' (OR) Run Group: 'Treated' (AND) Sample Type: 'Cell culture'

Q6. Search Experiments for 'POU5F1' Bait Genes for 'Embryonic stem' Cell Lines in AP-MS or 'Mus musculus' organisms that are not perturbated or endogenous cell cultures	Bait Gene = 'POU5F1" (OR) Cell Line = 'Embryonic stem" (AND) Experiment Type = "AP-MS" (OR) Organism = "Mus musculus" (AND) Perturbation = "Not Applicable" (OR) Sample Type = "Cell culture" (OR) Bait Type = "Endogenous"

Q7. Search Protein Expression Experiments or 'T-cells Cell Lines for Drosophila melanogaster organism for Tagged cell cultures not perturbated for APC Bait Genes and No vector control run groups	Experiment Type: 'Protein Expression' (OR) Cell Line: 'T-cells (Boom)' (AND) Organism: 'Drosophila melanogaster' (AND) Bait Type: 'Tagged' (AND) Sample Type: 'Cell culture' (OR) Perturbation: 'Not Applicable' (AND) Bait Gene: 'APC' (AND) Run Group: 'No Vector Control'

**Figure 12:**
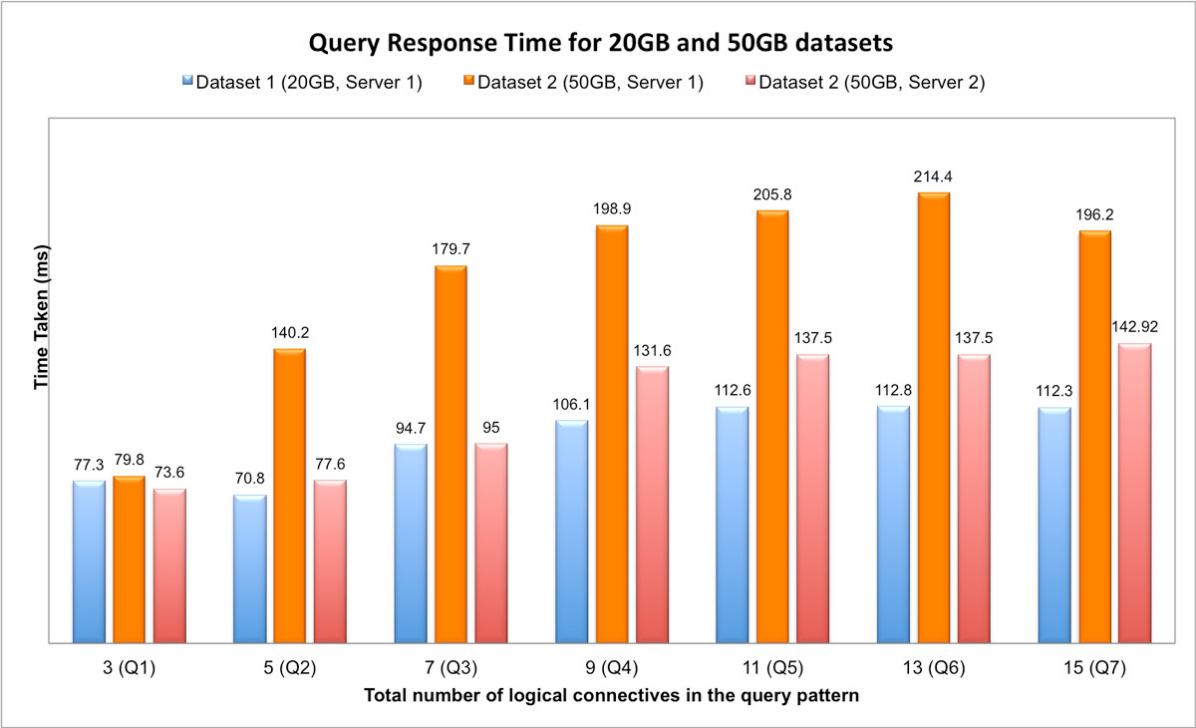
**Results for queries with increasing complexity over two datasets and two servers**. Performance evaluation of queries for increasing query complexity for the queries listed in Table 1.

Figure 12 shows the result of the evaluation, which is the average value of 5 consecutive query executions (with initial "cold cache"). The experiment was conducted on a server with 1.8 Ghz Intel Xeon processor and 24 MB cache size.

The results clearly show that the total time for increasingly complex queries is relatively stable over the two datasets. Although there is notable difference in performance between the 20 GB and 50 GB datasets for the same query (Figure 12), this issue can be effectively addressed by improving the hardware configuration of the server. For example, Figure 12 shows that simple upgradation of the cache size, from 512 KB to 24 MB, significantly improves the performance for all the queries. Hence, the total time for query execution in SemPoD is not expected to be a significant bottleneck for complex queries over large datasets.

## Discussion

The functionality of SemPoD query environment is primarily limited by the provenance and domain information modeled in the SysPro ontology. Hence, in the next phase of SemPoD development, we are modeling terms from additional metadata standards included in the MIBBI project. In addition, the SysPro ontology is being expanded to include concepts from GO and PRO to enable linking of genotype and protein data from external sources with CPB internal datasets. This allow researchers to query across genotype and phenotype data, including clinical information.

The manual mapping of SysPro ontology terms to the underlying database is an important challenge that can be addressed by creating semi-automated mapping techniques, which can define initial mappings through use of lexical matching and subsequently reviewed by researchers. Since automated schema mapping is still an open research problem, the involvement of researchers to manually verify the ontology-to-database mapping will ensure the accuracy of results in SemPoD. We plan to release the first version of the SemPoD codebase as a git hub open source project, which will allow other users and developers to review and use SemPoD in other 'omics center. Similarly, the first version of the SysPro ontology will be released for public use through listing at the National Center for Biomedical Ontologies (NCBO) [17]. We propose to define mappings between SysPro and other experiment metadata ontologies already listed at NCBO, including the Ontology for Biomedical Investigation (OBI) [18] and Experiment Factors Ontology (EFO) (derived from OBI) [19].

## Conclusions

Many researchers routinely use several different proteomics workflows to study biomedical problems. Studies may use different cohorts of patients, different cell lines or different techniques, but their value for biomedical discovery is significantly increased if researchers can query across these different studies as well as integrate with legacy data. The SemPoD platform is an ontology-driven intuitive query platform that leverages provenance metadata for effectively addressing these challenges. The SemPoD platform features four components to facilitate query composition using existing experiment metadata standard terms through an integrated ontology browser, a result browser, and a query manager to store queries for subsequent re-use or sharing with other researchers. The evaluation results for SemPoD, both in terms of positive user feedback and scalability for complex queries over increasing size of datasets, show that SemPoD can successfully meet the informatics requirements for large 'omics' research centers.

## Competing interests

The authors declare that they have no competing interests.

## Authors' contributions

CPJ designed the SemPoD architecture, implemented the query builder and ontology frameworks, integrated the Labkey system to SemPoD, and wrote the manuscript. MXZ implementated the query manager and validated the results of queries. SSS created the SysPro ontology and contributed to the writing of the manuscript. RME defined the usecases of the SemPoD framework and validated the results in the deployment phase. GQZ contributed to the designing the system architecture and writing the manuscript. All authors have read and approved the final manuscript.
